# Determination of cyanide in bamboo shoots by microdiffusion combined with ion chromatography–pulsed amperometric detection

**DOI:** 10.1098/rsos.172128

**Published:** 2018-04-18

**Authors:** Ming Ding, Kailiang Wang

**Affiliations:** The Research Institute of Subtropical Forestry, CAF Fuyang 311400, People's Republic of China

**Keywords:** microdiffusion, ion chromatography, pulsed amperometric detection, cyanide, bamboo shoots

## Abstract

A practical method for the determination of cyanide in bamboo shoots has been developed using microdiffusion preparation integrated with ion chromatography–pulsed amperometric detection (IC-PAD). Cyanide was released from bamboo shoots after Conway cell microdiffusion, and then analysed by IC-PAD. In comparison with the previously reported methods, derivatization and ion-pairing agent addition were not required in this proposed microdiffusion combined with IC-PAD method. The microdiffusion parameters were optimized including hydrolysis systems, temperature, time, and so on. Under the optimum conditions, the linear range of the calibration curve for cyanide was 0.2–200.0 µg kg^−1^ with satisfactory correlation coefficients of 0.9996 and the limit of detection was 0.2 µg kg^−1^ (*S/N* = 3). The spiked recovery range was from 92.8 to 98.6%. The intra-day and inter-day relative standard deviations of cyanide were 2.7–14.9% and 3.0–18.3%, respectively. This method was proved to be convenient in operation with high sensitivity, precision and accuracy, and was successfully applied in the determination of cyanide in bamboo shoot samples.

## 1. Introduction

Young and tender bamboo columns (known as bamboo shoots) are low in fats and cholesterol contents, but very high in potassium, carbohydrates and dietary fibres. Many nutritious and active materials such as vitamins, amino acids and antioxidants are present in bamboo shoots. As a kind of superior natural resource, bamboo shoots have been consumed for various food items in several countries. As reported in the literature [1–3], the overall bamboo shoots consumption worldwide is more than 2 million tons annually. The consumption of bamboo shoots is mainly concentrated in Southeast Asia. China has the largest bamboo industry producing approximately 1.3 million tons of fresh bamboo, while India is the second largest producer of bamboo shoots. However, despite the fact that tender bamboo shoots are not part of the traditional cuisine in Europe or North America, the popularity of Chinese restaurants worldwide gives an opportunity for people in many countries to taste the edible bamboo shoots. Moreover, with the trend of international trade, the consumption of bamboo shoots is widespread in Asia, Europe, North America, Oceania and Africa. Although bamboo shoots offer nutritional value, they contain potentially toxic compounds called cyanogenic glycosides (i.e. taxiphyllin), which can break down upon disruption of plant cells to form hydrogen cyanide (HCN) [4–6]. It is reported that fresh bamboo shoots contain cyanide as high as 25 mg kg^−1^, while cyanide content in dried, canned or boiled bamboo shoots is about 5.3 mg kg^−1^ [7]. The presence of HCN produces bitterness in the bamboo shoots, which limits the edible value. The acute lethal dose of cyanide for human beings is 0.5–3.5 mg kg^−1^. That is to say approximately 25–175 mg of free cyanide from bamboo shoots brings out a lethal dose for an adult man. Hence, cyanide in edible bamboo shoots must be detected at low concentrations according to its severe toxicity. However, the measurement of cyanide concentration in bamboo shoots is hindered by many obstacles. Thus, the development of an effective, sensitive and simple method to determine cyanide in bamboo is important.

As reported in the literature, several methods such as picrate and acid hydrolysis method [8–11], chemiluminescence [12], spectrophotometry [13–15], colorimetric method [16,17], sequential injection method [18], atomic-absorption spectrophotometry and ion chromatography [19,20] have been applied to measure cyanide. These methods involved the conversion of the analyte to HCN by a reaction with acid. The preparation process required distillation and had many measurement complications, including difficulty with oxidizers, requirement of high pH solutions, usage of toxic organic solvents and potential possibility of HCN leakage. Compared with the above-mentioned methods, ion chromatography (IC) is used extensively for various ionic compound analyses in the food and pharmaceutical industries. IC exhibits incomparable advantages because of its simple operation, fast analysis and being free of toxic solvents. Both DC amperometric detection and pulsed amperometric detection (PAD) have been used in IC methods for cyanide determination. In DC amperometry, the electrode surface is fouled by oxidation products because a constant voltage is applied. PAD uses a series of potentials which detect the compounds and then clean and restore the working electrode surface. The development and application of PAD provides IC with high selectivity and improves accuracy. These benefits have prompted the widespread application of PAD to IC. Recently, extensive efforts have been made for determining cyanide by IC-PAD method in matrices such as drinking water [19,21], liquor [20], cigarette mainstream smoke [22], ground water, sludge and soils [23]. However, this method is immature and has not been reported to determine cyanide in complicated bamboo shoot samples. This article first reports sensitive, fast, accurate determination of cyanide in bamboo shoot samples by IC-PAD after microdiffusion preparation.

Microdiffusion is a simple, quick and safe preparation method that can release cyanide from samples at room temperature by acidification without leakage. Since the pKa of HCN is 9.21, cyanide is supposed to be in the protonated form in a solution at pH 6.2. The HCN diffuses and then is absorbed by NaOH solution in the internal well. Feldstein & Klendshoj [24] first reported the application of microdiffusion for the extraction of cyanide in liquid samples such as blood and water. Microdiffusion preparation method has been reported for the treatment of available cyanide [25,26], nitrogen or chloride in real samples. To the best of our knowledge, this is the first time that microdiffusion preparation technique has been used for the isolation of cyanide in plant samples. In the present study, cyanide was released from bamboo shoots by Conway cell microdiffusion and analysed by IC-PAD. Compared with reported methods, the developed method is sensitive, time-saving and free of toxic organic solvents. Under the optimum conditions, the linear range of the calibration curve for cyanide was 0.2–200.0 µg kg^−1^ with satisfactory correlation coefficients of 0.9996 and the limit of detection (LOD) was 0.2 µg kg^−1^ (*S/N* = 3). The spiked recovery range was from 92.8% to 98.6% and the relative standard deviations (RSDs) were all less than 18.3%. This method was proved to be convenient in operation with high sensitivity, precision and accuracy, and was successfully applied in the determination of cyanide in bamboo shoots.

## 2. Experimental procedure

### 2.1. Instrumentation

All the chromatographic separations were carried out on a Thermo Fisher (Sunnyvale, CA, USA) ICS 3000 system, which was equipped with a DP dual gradient pump module, a 6-ports valve with a 25 µl sampling loop and DC detector. An ED50A electrochemical detector module that contained an electrochemical cell with a combination pH-Ag/AgCl reference electrode and a disposable silver working electrode was equipped with System 1 of the DC module. Poly(ether ether ketone) tubes were used to connect all the units. A Chromeleon 6.80 workstation (Sunnyvale, CA, USA) was used to control the ICS-3000 system and obtain data.

The chromatographic conditions used in the experiment were as follows: IonPac AG 7 (50 mm × 4 mm i.d. 10 µm; Thermo Fisher, USA) and IonPac AS 7 (250 mm × 4 mm i.d. 10 µm; Thermo Fisher, USA) columns; the columns were placed in the column oven at a temperature of 30°C; flow rate was set at 1.0 ml min^−1^; pulsed amperometric detection.

### 2.2. Reagents and materials

Free cyanide standard solution (50 µg g^−1^) was purchased from the National Institute of Metrology (China). All the other reagents used in this work were purchased from Sigma-Aldrich (Japan). Deionized water produced by Milli-Q (France) was used to prepare diluent, eluents and standards. Bamboo shoot samples were supplied by China Forestry Institute.

### 2.3. Microdiffusion preparation

**Figure 1.**
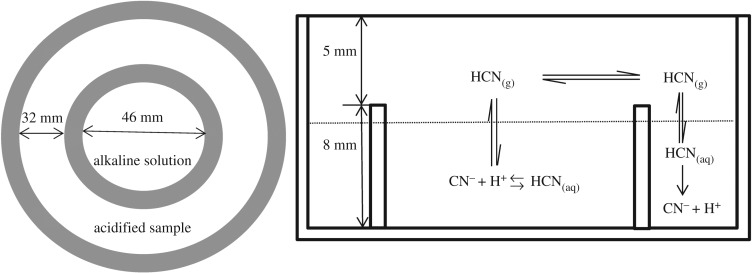
The procedure of microdiffusion preparation using Conway cell.

Bamboo shoots were solid samples with complicated matrix, so cyanide first needed to be isolated from bamboo shoots before analysis. The preparation was performed by microdiffusion using a Conway cell (shown in figure 1). A bamboo shoot was cut into small pieces and pounded. Then a sample of pulp (2.5 g) was placed in the external well with 1.0 ml cadmium chloride solution (50 g l^−1^), 10.0 ml potassium phosphate (pH = 6) and 1 ml β-glycosidase. The sample was acidified to release cyanide from the bamboo shoot in the form of HCN. Subsequently, 5 ml of sodium hydroxide solution (15 g l^−1^) was added to the internal well of Conway cell to absorb the gaseous HCN. The Conway cell was maintained at 40°C for 2 h. Afterwards, the absorption liquid was transferred to a 10 ml volumetric flask, diluted with water to the volume, filtered by microfiltration membrane, and then injected into the IC system and analysed directly. The analysis of the extracts was carried out using IC coupled with PAD.

### 2.4. Chromatographic conditions

Cyanide was separated in 12 min on an IonPac AG7 (50 mm × 4 mm) guard column and IonPac AS7 (250 mm × 4 mm) analytical column with isocratic elution. The elution was set as 50% 100 mM sodium hydroxide solution and 50% 500 mM sodium acetate (with 0.5% ethylene diamine, v/v) at a flow rate of 1.00 ml min^−1^ and column temperature was maintained at 30°C. The injection volume was 25 µl. Samples were detected by PAD which worked in AgCl mode with a combination pH-Ag/AgCl reference electrode and a disposable silver working electrode. The PAD waveform used was a three-potential waveform (E1, E2 and E3) previously optimized for disposable silver electrodes and cyanide (shown in table 1). E1 was used to detect cyanide, while E2 and E3 cleaned and restored the working electrode.

**Table 1.** Three-potential waveform standard for PAD.

time (s)	potential (V)	integration
0.00	−0.10	off
0.20	−0.10	on (start)
0.90	−0.10	off (end)
0.91	−1.00	off
0.93	−0.30	off
1.00	−0.30	off

## 3. Results and discussion

### 3.1. Chromatography

**Figure 2.**
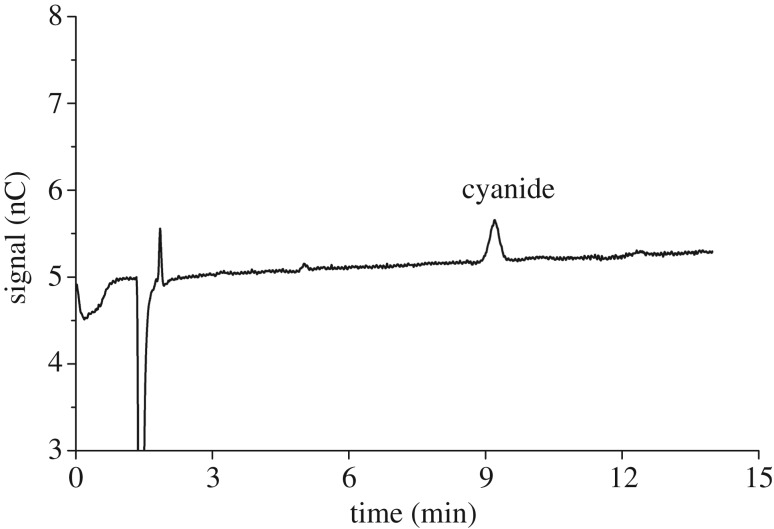
Typical chromatogram of free cyanide obtained by IC-PAD method under the following conditions. The elution was set as 50% 100 mM sodium hydroxide solution and 50% 500 mM sodium acetate (with 0.5% ethylene diamine, v/v). Flow rate: 1.0 ml min^−1^, injection volume: 25 µl, column temperature: 30°C, detection waveform table 1. Cyanide concentration: 7 µg kg^−1^.

Since the pKa of HCN is 9.21, the signal of cyanide is very weak in conductivity detection, which leads to very high detection limits. The development of DC amperometric detection and PAD provides IC with high selectivity and improves accuracy and sensitivity. In DC amperometry, a constant voltage is applied and cyanide is determined as response in current. However, DC amperometry has reported fouling problems over time. PAD uses a series of potentials that is frequently cycled rather than the single potential in DC amperometry. The waveform (−0.30 to −0.10 V) detects cyanide and cleans the working electrode surface. In order to validate IC-PAD method in practical use for the complicated bamboo shoot samples, it is necessary to evaluate interferences during analysis procedure. As shown in figure 2, common interfering ions (S^2−^, Cl^−^, ${\text{SO}}_{4}^{2-}{\text{ and SO}}_{3}^{2-}$) do not interfere with free cyanide. Hence, IC method with PAD detection is sensitive, selective, fast and robust for cyanide analysis.

### 3.2. Microdiffusion optimization

#### 3.2.1. The effect of hydrolysis system

The distillation method is considered as a classical technique and is widely applied for the preparation of cyanide. This method requires high temperature and has many measurement complications, including difficulty with high-pH solutions, low recovery and leakage of the hazardous HCN product. Compared with the above-mentioned method, the conditions of microdiffusion are much more moderate. Cyanide exists in bamboo shoots in a form of cyanogentic glycosides so that tartaric acid (pH = 4) or phosphate buffered solution (pH = 6) are enough to break the cyanogentic glycoside form in microdiffusion. During the procedure of microdiffusion, metal ions in bamboo shoots would affect the release of cyanide by complexing action. Hence, a small amount of complexing agent (e.g. zinc acetate, cadmium chloride, stannous chloride or EDTA) was added to eliminate the metal ion interference.

**Figure 3.**
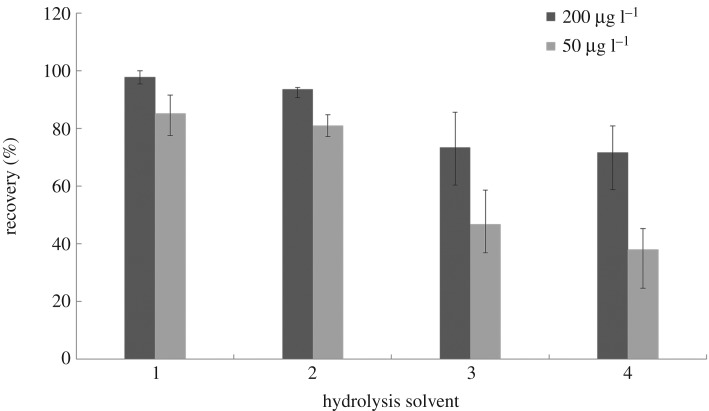
Comparison of different hydrolysis systems for cyanide in bamboo shoots (*n* = 6). (1) Complexing agent: 1 ml zinc acetate (100 g l^−1^), acidifier:10 ml tartaric acid (pH = 4); (2) complexing agent: 1 ml cadmium chloride (50 g l^−1^), acidifier: 10 ml phosphate buffered solution (pH = 6); (3) complexing agent: 1 ml stannous chloride (50 g l^−1^) and 5 ml copper sulfate (200 g l^−1^), acidifier: 10 ml 85% phosphoric acid buffer (pH < 2); (4) complexing agent: 1 ml EDTA solution, 1 ml 20% copper sulfate and 0.2 ml stannous chloride solution, acidifier: 10 ml 85% phosphoric acid buffer (pH < 2). Other microdiffusion conditions: 40°C, 4 h.

In this work, different hydrolysis systems were investigated in detail to achieve the best efficiency. Complexing agent was added to the samples during microdiffusion to avoid complexing action between cyanide and metal ions. The influence of several complexing agents (zinc acetate, cadmium chloride, stannous chloride or EDTA) on the final recovery was examined. The pH of acid was varied in different hydrolysis systems. The results are depicted in figure 3. According to the results, the best hydrolysis system for bamboo shoots was using tartaric acid (pH = 4) as the acid with zinc acetate as the complexing agent.

#### 3.2.2. The effect of temperature and time

**Figure 4.**
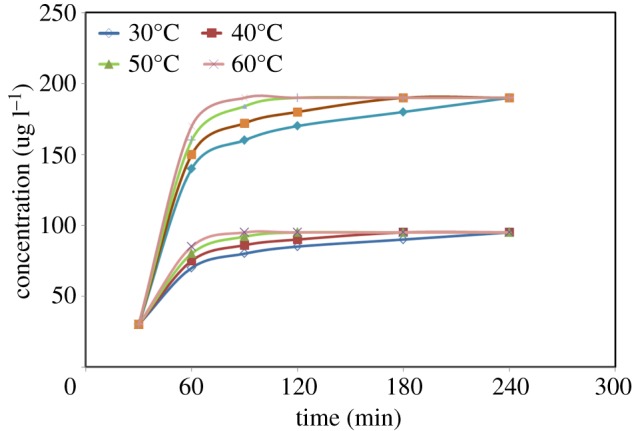
Effect of equilibration time (0–240 min) and microdiffusion temperature (30–60°C) on the extraction efficiency. Complexing agent: 1 ml cadmium chloride (50 g l^−1^); acidifier: 10 ml phosphate buffered solution (pH = 6).

Though HCN is a volatile gas with a boiling point of 25.6°C, it has good solubility in water. Hence, the optimization of temperature and time in microdiffusion is necessary to achieve the maximum efficiency. As shown in figure 4, the effects of temperature (in the range of 30–60°C) and time (in the range of 0–240 min) were investigated in detail. According to figure 4, the efficiency increases significantly at the first 120 min, then increases slowly and becomes stable finally. It is worth mentioning that with different temperature, the microdiffusion process was different. With the increase of the temperature from 30°C to 50°C, the microdiffusion process duration could be reduced dramatically from 240 min to 120 min. This effect was due to the increase in diffusion coefficient of hydrocyanic acid with temperature elevated. The microdiffusion system with higher temperature improved the mass transfer rate and rapidly achieved equilibrium. In conclusion, the microdiffusion process was conducted at 50°C for 120 min in the subsequent experiments.

#### 3.2.3. Comparison between acid hydrolysis and enzymolysis

**Figure 5.**
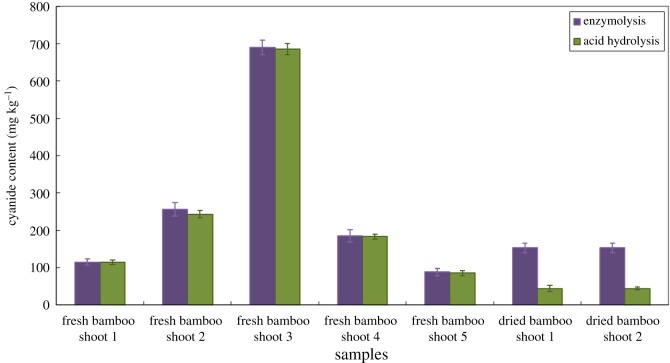
Comparison of enzymatic hydrolysis and acid hydrolysis cyanide in fresh bamboo shoots and dried bamboo shoot products.

Bamboo shoots contain potentially toxic compounds called cyanogenic glycosides, which break down upon disruption of the plant cells to form HCN. HCN exists in bamboo shoots in the form of cyanogenic glycosides. In this paper, the efficiency of acid hydrolysis and enzymolysis was studied and compared in seven bamboo samples. As shown in figure 5, acid hydrolysis and enzymolysis gave comparable results for fresh bamboo shoot samples. This is because there are β-glycosidases existing in fresh bamboo cells. As a result, without adding β-glycosidases, HCN also can be released from cyanogenic glycosides. According to figure 5, the efficiency of microdiffusion with acid hydrolysis and enzymolysis differs widely in dried bamboo shoots. As β-glycosidases are inactivated in dried bamboo shoots, they cannot break the glucosidic bond in cyanogenic glycosides effectively, resulting in low contents of HCN products. In this case, it is necessary to add exogenous active β-glycosidases into the dried bamboo shoot samples to fully release HCN. With the addition of β-glycosidases, the contents of HCN in dried bamboo shoots are 3.4–12.7 times that of acid hydrolysis.

### 3.3. Method validation

The analytical performance of the microdiffusion coupled with IC-PAD method was evaluated under the optimized experimental conditions described above. To measure the linearity of the method, the calibration curve was plotted at six concentration levels of the analytes. The following concentration levels for cyanide in standard solutions were studied: 0.2, 0.5, 2, 5.0, 10.0, 20.0, 50.0, 100.0 and 200.0 μg kg^−1^. The calibration curve was *y* = 73.26*x* + 11.49 with good linearity (*R* = 0.9996). LOD was calculated at a signal-to-noise (*S/N*) ratio of 3 and was found to be as low as 0.2 µg kg^−1^ for cyanide in bamboo shoots. Limit of quantitation (LOQ) was calculated based on a signal-to-noise ratio of 10 and was found to be 0.5 µg kg^−1^ for this method. Regression equation, correlation coefficient (*R*^2^), linear range, LOD and LOQ are summarized in table 2.

**Table 2.** Linear range, correlation coefficient, LOD and LOQ of cyanide.

compound	linear range (µg kg^−1^)	regression equation	Correlation coefficient (*R*)	LOD (µg kg^−1^)	LOQ (µg kg^−1^)
cyanide	0.2–200.0	*Y* = 73.26*X* + 11.49	0.9996	0.2	0.5

Subsequently, to evaluate the precision and the accuracy of the present method, the RSD and recovery were investigated by 2.5 g of spiking blank bamboo shoot samples with ultra-low (5 µg kg^−1^), low (50 µg kg^−1^), moderate (100 µg kg^−1^) and high (200 µg kg^−1^) levels of cyanide standards. Samples were routinely pretreated by microdiffusion prior to IC-PAD. As free cyanide molecules might not be stable over time, both intra-day and inter-day precision tests were investigated in the work. The intra-day and inter-day RSDs of free cyanide were 2.7–14.9% and 3.0–18.3%, respectively. The recoveries of cyanide in spiked samples varied from 92.8 to 98.6%. The RSDs and recoveries are listed in table 3, which indicates acceptable reproducibility and high precision of this method for cyanide analysis in bamboo shoot samples.

**Table 3.** Recoveries of standard addition and RSD for cyanide (*n* = 6).

analyte	added (μg kg^−1^)	recoveries (%)	intra-day RSD (%)	inter-day RSD (%)
cyanide	5	81.6	14.9	18.3
	50	92.8	11.7	15.2
	100	97.9	6.4	8.7
	200	98.6	2.7	3.0

### 3.4. Application to real samples

**Figure 6.**
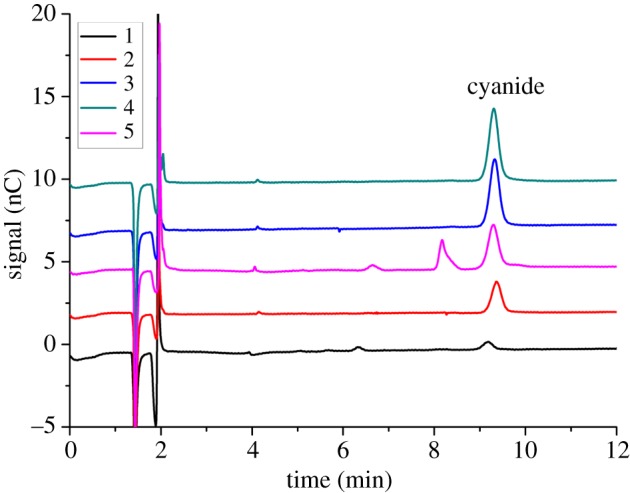
Chromatograms of cyanide in a variety of bamboo shoot samples (five samples) by microdiffusion coupled with IC-PAD method. These samples were treated with microdiffusion process. The separation conditions are as follows: isocratic elution was set as 50% 100 mM sodium hydroxide solution and 50% 500 mM sodium acetate (with 0.5% ethylene diamine, v/v); flow rate: 1.0 ml min^−1^; injection volume: 25 µl; column temperature: 30°C; detection waveform table 1.

The proposed microdiffusion coupled with IC-PAD method was applied to the determination of cyanide in a variety of bamboo shoot samples. For the samples tested, cyanide was detected in all the samples with the contents ranging from 53 to 690 µg kg^−1^. The obtained results indicated that each of the bamboo samples has a different cyanide level. The detailed results of cyanide in tested samples are summarized in table 4. Some representative chromatograms of the examined samples are given in figure 6. In addition, the results of the proposed method were compared with those of distillation–photometric method. As shown in table 4, the contents of cyanide obtained by the proposed method are comparable to those of distillation–photometric method. Besides, the LOD of the proposed method (0.2 µg kg^−1^) is much lower than that of the distillation–photometric method (15 µg kg^−1^) reported in the literature. By comparison, it is clear that the proposed microdiffusion coupled with IC-PAD method is much more sensitive for measuring free cyanide and is a demonstrably easy-to-operate, simple and reliable technique for the determination of cyanide in bamboo shoot samples.

**Table 4.** Comparison of the results of actual samples between the proposed method and distillation–photometric method.

	cyanide content (μg kg^−1^)	
sample	proposed method	distillation–photometric method
ray bamboo shoot^a^	115	115
moso bamboo shoot^a^	256	243
*Dendrocalamus latiflorus*^a^	690	685
fresh bamboo shoot 1	185	183
fresh bamboo shoot 2	88	85
dried bamboo shoot 1	53	44
dried bamboo shoot 2	53	44

^a^These bamboo shoots are fresh samples.

## 4. Conclusion

At present, the analytical procedures available for the measurement of cyanide content in solid samples such as bamboo shoots are inadequate and probably inaccurate. The errors are mainly introduced during the sample preparation and collection steps, which lead one to overestimate or underestimate the recovery of cyanide. In this case, microdiffusion coupled with IC-PAD method was proposed for the determination of cyanide in bamboo shoot samples. Metal ions are often present in bamboo shoots and can bond to cyanide by complexation reaction. The interference of metal ions is avoided by adding complexing agent to the bamboo shoot samples. As a pretreatment method, microdiffusion is more advantageous and effective than reported distillation techniques for bamboo shoot samples. In this study, microdiffusion optimization and method performance were discussed. The results showed that the proposed method had a wide linearity from 0.2 to 200.0 µg kg^−1^ and was sensitive enough to measure cyanide at low level (LOD = 0.2 µg kg^−1^) with good recovery. The method exhibited high resolution and the analysis time of samples was 12 min or less in IC-PAD. This work indicates that the optimized microdiffusion coupled with IC-PAD method is capable of analysing cyanide in bamboo shoot samples and may have potential applications for cyanide analysis in liquid and other solid samples.

## Data accessibility

This article does not contain any additional data.
